# Acute haemodynamic changes during haemodialysis do not exacerbate gut hyperpermeability

**DOI:** 10.1042/BSR20181704

**Published:** 2019-04-12

**Authors:** Jonathan Wong, Kaatje Lenaerts, Dennis M. Meesters, Steven W.M. Olde Damink, Hans M.H. van Eijk, Enric Vilar, Ken Farrington

**Affiliations:** 1 Department of Postgraduate Medicine, Hertfordshire, University of Hertfordshire, U.K.; 2 Department of Renal Research, Stevenage, East and North Hertfordshire NHS Trust, U.K.; 3 Department of Surgery, NUTRIM School for Nutrition and Translational Research in Metabolism, Maastricht University, Maastricht, The Netherlands

**Keywords:** Haemodialysis, Intestinal permeability, Uraemia

## Abstract

Introduction: The gastrointestinal tract is a potential source of inflammation in dialysis patients. *In vitro* studies suggest breakdown of the gut barrier in uraemia leading to increased intestinal permeability and it is hypothesised that haemodialysis exacerbates this problem due to mesenteric ischaemia induced by blood volume changes during treatment.

Method: The effect of haemodialysis on intestinal permeability was studied in ten haemodialysis patients and compared with five controls. Intestinal permeability was assessed by measuring the differential absorption of four orally administered sugar probes which provides an index of small and whole bowel permeability. A multi-sugar solution (containing lactulose, rhamnose, sucralose and erythritol) was orally administered after an overnight fast. Plasma levels of all sugar probes were measured hourly for 10 h post-administration. In haemodialysis patients, the procedure was carried out twice — once on a non-dialysis day and once immediately after haemodialysis.

Results: Area under curve (AUC) for lactulose:rhamnose (L:R) ratio and sucralose:erythritol (S:E) ratio was similar post-dialysis and on non-dialysis days. AUC for L:R was higher in haemodialysis patients compared with controls (0.071 vs. 0.034, *P*=0.001), AUC for S:E ratio was not significantly different. Levels of lactulose, sucralose and erythritol were elevated and retained longer in haemodialysis patients compared with controls due to dependence of sugars on kidney function for clearance.

Conclusion: We found no significant acute changes in intestinal permeability in relation to the haemodialysis procedure. Valid comparison of intestinal permeability between controls and haemodialysis patients was not possible due to the strong influence of kidney function on sugar levels.

## Introduction

Chronic systemic inflammation is highly prevalent in patients with advanced kidney disease. It is a strong cardiovascular risk factor and is associated with other complications including malnutrition, cachexia, anaemia and early mortality [1]. The pathophysiology of chronic inflammation in the dialysis population is not well understood although it is likely to be due to multiple factors including increased oxidative stress [2], underlying pro-inflammatory conditions and chronic subclinical infection related to dialysis access [3]. The gastrointestinal tract is increasingly being recognised to be a major source of chronic inflammation in the dialysis population [4,5].

Development of renal impairment leads to alteration in the intestinal microbiome due to changes in the biochemical milieu of the alimentary tract which are known to influence and damage gut barrier function [6,7]. The intestinal barrier acts as a semipermeable membrane for the selective absorption of essential dietary nutrients, electrolytes and water from the intestinal lumen while preventing translocation of harmful microbial products and pathogens into circulation [8]. The intestinal barrier consists of epithelial cells which regulate transcellular transport of solutes and intercellular junctional complexes or ‘tight junctions’ which seal the spaces between epithelial cells regulating the entry of luminal contents through these paracellular routes [9]. These tight junctions consist of adhesive transcellular proteins (occludin and claudin families) and actin-binding cytosolic proteins (zonula occludens family) [5].

A large number of *in vitro* and observational studies in animal and humans with chronic kidney disease (CKD) strongly suggest that there is breakdown of intestinal barrier function [reviewed in [4,5,10]]. Studies in non-dialysed CKD patients and uraemic rats demonstrate increased absorption of large-sized polyethylene glycols indicating increased intestinal permeability or ‘leakiness’. *In vivo* work in animals has demonstrated marked loss of occludin, claudin and zona occludens from the intestinal tracts of CKD subjects [11]. Breakdown in intestinal barrier function has been hypothesised to contribute to systemic inflammation due to the translocation of large quantities of bacterial components across the ‘leaky’ intestinal wall and entry into the blood circulation [12–14].

Postulated mechanisms of intestinal barrier damage include direct disruption of tight junctions caused by uraemic toxins [15,16] and in haemodialysis patients, ultrafiltration during treatment may also cause hypotension leading to bowel ischaemia [17–19], exacerbating uraemia-induced intestinal barrier dysfunction [5]. However, intestinal permeability has not been measured *in vivo* in haemodialysis patients and direct intestinal permeability changes, induced by the acute effect of haemodialysis, have not been studied. If haemodialysis-induced gut permeability were to be demonstrated, interventions could be targeted at the haemodialysis procedure to minimise this effect.

The lack of studies on intestinal permeability in dialysis patients is likely due to the difficulties in applying the conventional method of measuring intestinal permeability in this population. Intestinal permeability *in vivo* is measured by determining the urinary excretion of orally administered test substances. These test substances typically consist of a disaccharide and a monosaccharide, the ratio of the urinary concentrations of both providing a specific index of intestinal permeability [20]. Since a significant proportion of dialysis patients are anuric, measurement of intestinal permeability using this method is not possible. A sensitive assessment of sugars based on liquid chromatography in combination with mass spectrometry (LC-MS) has been developed which allows measurement of sugars in plasma [21–23].

The purpose of the present study was to determine the acute effect of haemodialysis on intestinal permeability. Intestinal permeability in haemodialysis patients was also compared with healthy controls.

## Methods

### Ethical approval

The present study was granted ethical approval by the East of England – NHS Cambridge East Research Ethics Committee, reference 15/EE/0379. Informed written consent was obtained from all participants prior to the study. Experimental work was carried out in accordance with the World Medical Association Declaration of Helsinki.

### Participant characteristics

Ten patients on maintenance haemodialysis and five healthy volunteers were recruited for the present study. Haemodialysis patients were medically stable with no active illness at the time of the study. Exclusion criteria were positive HIV or hepatitis B/C status, active gastrointestinal symptoms or disease, liver disease and history of previous bowel surgery.

### Gut permeability testing

The classical assays for gut permeability are usually based on a difference in intestinal absorption of two supplied sugars, usually a disaccharide and monosaccharide. A ratio of the concentration of disaccharide over the monosaccharide recovered in the urine or plasma following oral administration is used as an index of intestinal permeability. In states of increased intestinal permeability, there is a relative increase in the absorption of the larger disaccharide molecule due to increased paracellular transport leading to a higher disaccharide to monosaccharide ratio. A multi-sugar solution consisting of lactulose, rhamnose, sucralose and erythritol was used in the present study for the assessment of intestinal permeability. Lactulose (disaccharide) and rhamnose (monosaccharide) was used as a marker for small intestinal permeability since they are degraded by the microbiota in the colon [24,25]. Sucralose (disaccharide) and erythritol (monosaccharide) was used as a marker for whole gut permeability since they resist colonic bacterial fermentation [26]. Sugar concentrations in plasma were measured at repetitive time points using LC-MS as described below. The calculated lactulose:rhamnose ratios (L/R) were used to assess small intestinal permeability and sucralose:erythritol ratios (S/E) for whole gut permeability. Plasma concentrations of sugars were measured over a 10-h period. The optimum time period to assess sugar ratios derived from plasma have not yet been established, although for sugar ratios derived from urinary fractions, 0–5 and 5–24 h appear to best reflect small and large intestine permeability respectively [27]. Therefore for comparison of small intestinal permeability between haemodialysis patients and healthy controls, L/R ratios were assessed for 5 h after sugar ingestion, for whole bowel permeability S/E ratios were assessed for 10 h. However, since sugar profiles in haemodialysis patients have not been studied previously and may be different from healthy volunteers, comparison of L/R and S/E ratios between dialysis and non-dialysis days were studied during the early period (0–5 h post-sugar ingestion), late period (5–10 h post-ingestion) and over the whole 10-h study period.

### Study design and sampling

On the day before and during the test days, all participants were asked to avoid intense physical exercise and consumption of any sweets, confectionery, desserts, sugar-free chewing gum and non-steroidal anti-inflammatory drugs. Products containing erythritol or sucralose were avoided. Participants were tested after an overnight fast, a baseline blood sample was collected from either a cannula inserted in the forearm or from an existing tunnelled dialysis catheter (for haemodialysis patients) using standard aseptic technique. A multi-sugar solution consisting of 1 g lactulose (TEVA UK Limited, 3.35 g/5 ml), 1 g rhamnose (Danisco Sweeteners), 1 g sucralose (Brenntag, Netherlands) and 1 g erythritol (Danisco Sweeteners) dissolved in 100 ml water was orally administered. Following ingestion, blood samples were collected hourly for 10 h. At each sampling point, blood was collected into EDTA tubes and centrifuged at 2300×***g*** for 15 min to obtain plasma. Plasma samples were aliquoted, frozen and stored at −80°C. Subjects were allowed to eat 2 h after ingestion of sugars although all sweets, products containing sweeteners were avoided throughout the study period. Participants were not restricted in position or mobility although subjects were not allowed to carry out intense exercise during the study period.

For haemodialysis patients, intestinal permeability was measured on a non-dialysis day and on a haemodialysis day. Both study days were carried out within 1 week of each other. Testing was randomised such that half of the cohort had initial permeability measurements carried out on a non-dialysis day followed by repeat testing carried out on a dialysis day. The remainder of the cohort were tested in reverse order. For intestinal permeability measurements performed on a haemodialysis day, the study commenced immediately at the end of the dialysis session. The concentration of sugars post dialysis were corrected for dialysis-induced changes in blood volume by multiplying the concentration of the sugar after dialysis with the ratio of serum albumin before and after dialysis at each time point [28].

### Analysis of sugars

Measurement of sugar probes in plasma was carried out using isocratic ion-exchange high performance liquid chromatography (Model PU-1980 pump, Jasco Benelux, Netherlands) and mass spectrometry (Model LTQ-XL, Thermo Electron, Netherlands) [21]. Three hundred microliters of plasma was transferred into Eppendorf cups containing a 3000-Da cut-off filter (Amicon Ultra 0.5 ml 3K, Millipore) to remove plasma proteins. The filter cups were centrifuged for 30 min at 11000***g*** at 4°C to obtain clear plasma filtrate. The plasma filtrate was transferred into 300-µl glass insert, spring loaded in a 4-ml WISP style vial (Waters, Milford, U.S.A.) and placed into a Peltier chilled Gilson 233XL sample processor (Gilson, U.S.A.). Chromatographic separation was based on isocratic elution of individual sugars probes on an IOA-1000 9 μm cation-exchange column (300 mm × 7.8 mm ID; Illinois), mounted in a Mistral column oven (Separations, Netherlands) at 30°C. An aqueous solution of 20 mmol/l formic acid and 10 mmol/l trichloroacetic acid was delivered using a Model PU-1580 HPLC pump (Jasco Easton, Maryland) at a flow rate of 0.225 ml/min. Samples and standards were injected using a Model 233XL sample processor with Peltier chilled sample storage compartments [10°C], equipped with a 20-µl sample loop. After separation, the column effluent was mixed with 30 mmol/l ammonia in 20% methanol/water (v/v) delivered by an additional Model PU 980 pump to allow the formation of ammonium adducts. MS detection was performed using a model LTQ XL (Thermo Fisher Scientific, Massachusetts) equipped with an ion-Max electrospray probe. The mass spectrometer was operated in positive mode. Spray voltage was 4.8 kV. Sheath and auxiliary gas were 99 and 30 units respectively with capillary temperature of 220°C. The system was set to a mass range of 125–460 Da in full-scan enhanced mode.

### Statistical analysis

Statistical analysis was performed using GraphPad Prism and SPSS Statistics software. Sugar concentrations were plotted against time for each participant and visually inspected for outliers. Outliers were excluded from the analysis. Area under curve (AUC) was calculated for each sugar, L/R and S/E ratios for all subjects. Derived AUC data for sugars were not normally distributed therefore comparison of AUC between haemodialysis patients and controls were evaluated using Mann–Whitney U-test and comparison of sugar profiles between haemodialysis and non-haemodialysis days were evaluated using Wilcoxon’s signed rank test.

## Results

Patient and healthy control characteristics are displayed in Table 1. Haemodialysis patients and controls were similar in terms of age, weight and body mass index. All haemodialysis patients had stable blood pressures before, during and after dialysis, ultrafiltration requirement and rate was moderate. Mean ultrafiltration volume and rate was 1.91 l and 6.3 ml/kg/h respectively. Forty percent of patients were anuric. The median dialysis vintage was 0.85 years. Haemodialysis patients were on several medications as displayed in Table 2, no patients were on antibiotics or non-steroidal anti-inflammatory drugs medications at the time of the study. Some patients were on medications that could affect gut motility including opiates (*n*=3), steroids (*n*=1). Four patients were on proton pump inhibitors at the time of the study which have been reported to increase upper gastrointestinal tract permeability [29].

**Table 1:** Subjects’ clinical and demographic data

Variable	HD patients (*n*=10)	Controls (*n*=5)	*P*
Age (years)	49 ± 3.7	46.2 ± 4.4	0.82
Weight (kg)	84 ± 7	66 ± 4.2	0.11
Height (m)	1.72 ± 0.03	1.6 ± 0.02	0.03*
BMI	28.1 ± 1.9	25.5 ± 0.98	0.36
Charlson Comorbidity index	3 [IQR 2.5]		
Ultrafiltration volume (l)	1.91 ± 0.3		
Ultrafiltration rate (ml/kg/h)	6.3 ± 1.3		
Dialysis session time (min)	230 [IQR 30]		
Pre-dialysis BP (mmHg)	149/76		
Post-dialysis BP (mmHg)	130/78		
Kt/V	1.32 ± 0.1		
Residual urea clearance (ml/min)	1.1 [IQR 2.8]		
Proportion with no residual kidney function (%)	40%		
Proportion with tunnelled dialysis catheter (%)	40%		
Dialysis vintage (years)	0.85 [IQR 2.6]		

Abbreviations: BMI, body mass index; BP, blood pressure; HD, haemodialysis; IQR, interquartile range. *denotes statistical significance *P*<0.05.

**Table 2:** Medications used by haemodialysis patients

Medication	Number of patients (*n*=10)
**Vitamin D analogues**	
Alfacalcidol	7
**Calcimimetic**	
Cinacalcet	2
**Antihypertensives**	
Calcium channel blockers	6
β-blockers	3
Diuretics	4
α-blockers	3
Angiotensin receptor blockers	1
Vasodilators	1
**Opiates** (codeine phosphate)	3
**Proton pump inhibitors**	4
**Hypoglycaemic agents**	
Insulin	2
**Phosphate binders**	9
**Antidepressants**	
Selective serotonin reuptake inhibitor	3
**Immunosuppressive medications**	
Calcineurin inhibitors	1
Steroids	1
**Statins**	3
**Antiplatelets**	
Aspirin (omitted 24 h prior to study)	3
Ticagrelor	1
**Others**	
Gabapentin	5
Allopurinol	3
Montelukast	1
Quinine sulphate	5

### The effect of haemodialysis on intestinal permeability

**Figure 1:**
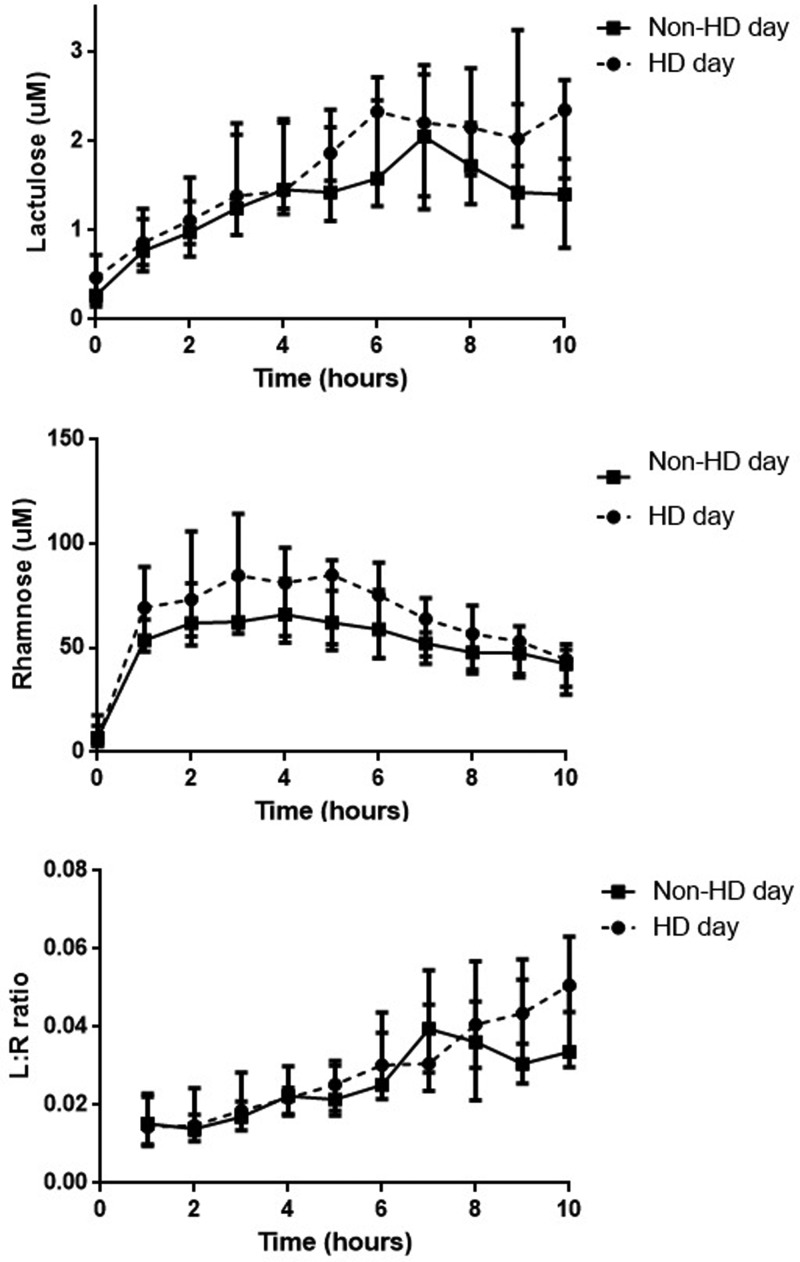
Plasma concentrations of lactulose, rhamnose and L/R ratios on a non-dialysis day and after haemodialysis treatment

**Figure 2:**
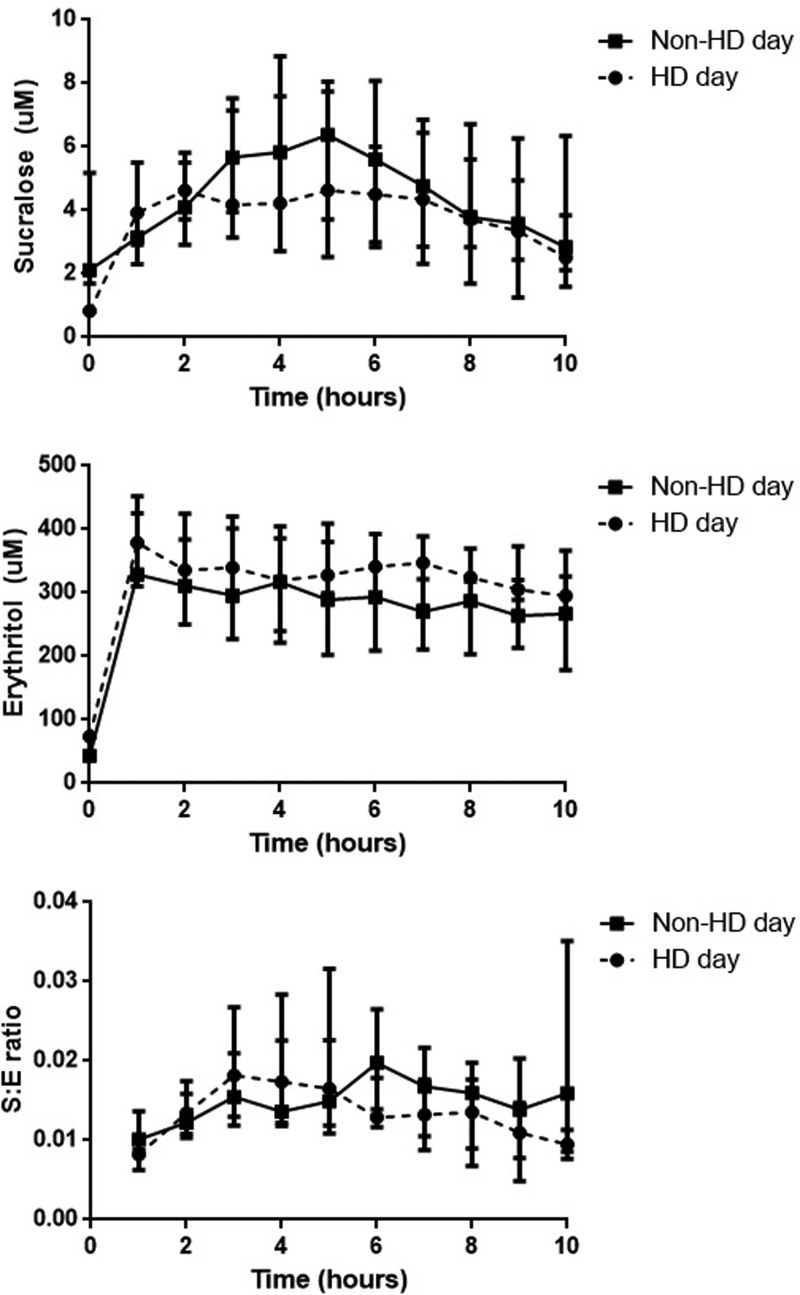
Plasma concentrations of sucralose, erythritol and S:E ratios on a non-dialysis day and after haemodialysis treatment

AUC of studied sugar probes were compared in haemodialysis patients on a non-dialysis day and immediately after dialysis to determine the effect of the haemodialysis procedure on intestinal permeability. During the early phase (0–5 h) AUC for rhamnose was significantly higher after haemodialysis treatment however, the lactulose levels and L/R ratio were not significantly different (Figure 1 and Table 3). There were no significant differences in lactulose, rhamnose or L/R ratios between dialysis and non-dialysis days assessed in the late period (5–10 h) or over the whole study period (0–10 h). Similarly, there were no significant differences in AUC of sucralose, erythritol and S/E ratio between non-dialysis days and after haemodialysis treatment (Figure 2 and Table 2).

**Table 3:** AUC and plasma concentrations of sugar probes on non-dialysis days and after haemodialysis treatment

	Non-HD day (*n*=10)^†^	HD day (*n*=10)^†^	*P*
**Early period (0–5 h)**			
Lactulose	5.48 [4.72–8.01]	5.48 [5.01–8.84]	0.72
Rhamnose	273.1 [234.3–393.5]	308.5 [273.3–464.7]	0.01*
L:R ratio	0.073 [0.063–0.112]	0.078 [0.064–0.086]	0.72
Sucralose	26.6 [14.5–29.8]	19.9 [17.1–29.9]	0.95
Erythritol	1411 [1098–1788]	1635 [1420–1905]	0.26
S:E ratio	0.055 [0.047–0.09]	0.067 [0.044–0.076]	0.37
**Late period (6–10 h)**			
Lactulose	6 [5.29–10.86]	8.43 [5.91–10.47]	0.51
Rhamnose	177.4 [127.7–230.7]	217 [156.6–278.3]	0.17
L:R ratio	0.146 [0.098–0.19]	0.157 [0.132–0.2]	0.96
Sucralose	16 [7.4–26.2]	15.3 [10.3–22.5]	0.86
Erythritol	1021 [779–1277]	1333 [1092–1474]	0.05
S:E ratio	0.067 [0.029–0.081]	0.051 [0.038–0.062]	0.59
**Whole study period (0–10 h)**			
Lactulose	12.62 [11.08–21.17]	19.39 [12.58–21.17]	0.45
Rhamnose	541.2 [428.9–702.7]	634.5 [497.7–805.7]	0.09
L:R ratio	0.242 [0.188–0.338]	0.274 [0.223–0.314]	0.96
Sucralose	48.5 [24.6–63.0]	42.8 [30.4–56.7]	0.59
Erythritol	2800 [2076–3514]	3131 [2943–3743]	0.14
S:E ratio	0.15 [0.087–0.194]	0.136 [0.094–0.153]	0.31

Abbreviation: HD, haemodialysis. *denotes statistical significance, *P*<0.05. ^†^One haemodialysis patient with high baseline plasma concentrations of sucralose was excluded and not included in analysis with regards to sucralose, erythritol, and S:E ratios. Data shown are median and interquartile range.

### Comparison of intestinal permeability between haemodialysis patients and healthy controls

#### Small bowel permeability

**Figure 3:**
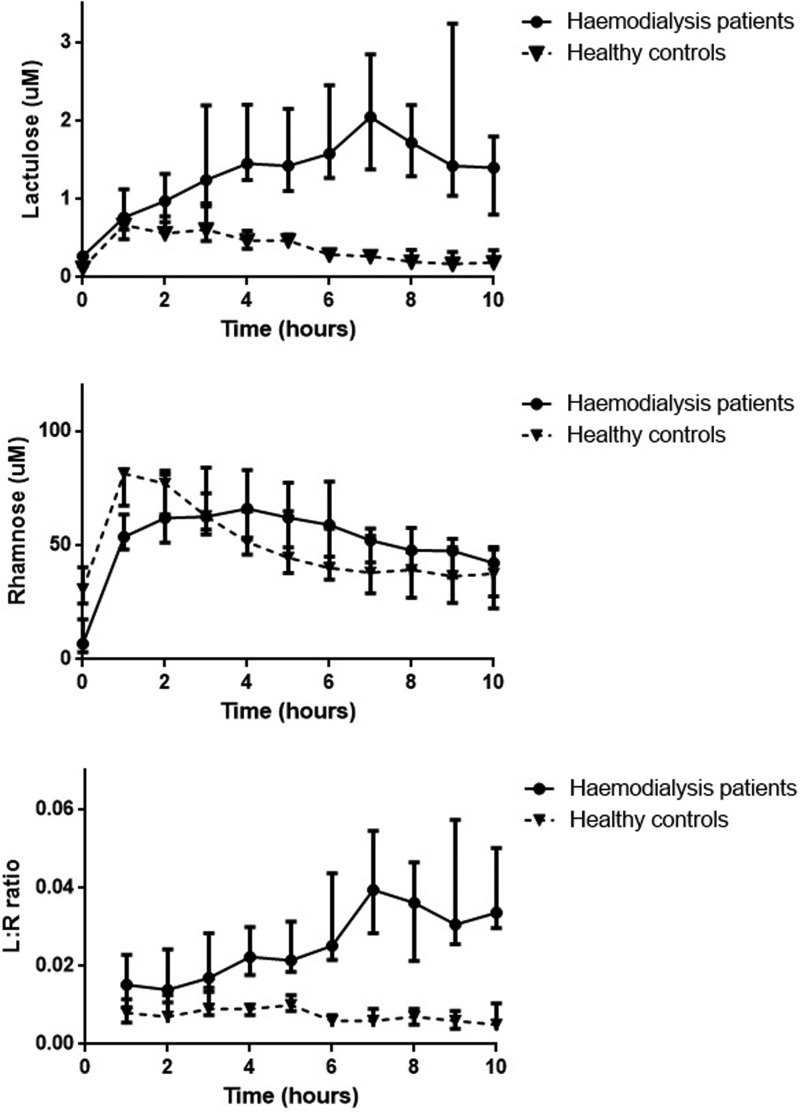
Plasma concentrations of lactulose, rhamnose and L:R ratio in haemodialysis patients and healthy controls

Small bowel permeability was assessed by comparing the differential absorption of lactulose and rhamnose. Plasma levels of lactulose increased rapidly after ingestion for both haemodialysis patients and controls (Figure 3), for healthy controls lactulose levels peaked after 1 h, for haemodialysis patients lactulose levels continued to rise throughout the study period and were approximately three times greater than peak levels reached by healthy controls (2.05 vs. 0.67 μM, *P*=0.002). AUC of lactulose was significantly higher than healthy controls (5.27 vs 2.66, *P*=0.001). Plasma levels of rhamnose peaked at 1 h after ingestion for healthy controls, for haemodialysis patients rhamnose concentration reached peak levels later at approximately 4 h after ingestion, the peak concentration reached was similar for both groups. There was no significant difference in AUC of rhamnose levels between both groups (283 vs. 307.4, *P*=0.679). AUC for L/R ratios were significantly higher in haemodialysis patients compared with healthy controls (0.071 vs. 0.034, *P*=0.001) (Figure 3 and Table 4).

**Table 4:** AUC and median plasma concentrations of sugar probes in haemodialysis patients and healthy controls

	Healthy controls (*n*=5)	HD patients (*n*=10)^†^	P
Lactulose	2.66 [2.16–3.31]	5.27 [4.43–7.74]	0.001*
Rhamnose	307.4 [267.4–353.8]	283 [242.2–380.4]	0.679
L:R ratio	0.034 [0.03–0.048]	0.071 [0.058–0.11]	0.001*
Sucralose	16.5 [15.2–34.2]	48.5 [24.6–63]	0.019*
Erythritol	1150 [1111–1732]	2800 [2076–3514]	0.001*
S:E ratio	0.13 [0.12–0.21]	0.15 [0.09–0.19]	0.797

Abbreviation: HD, haemodialysis. *denotes statistical significance, *P*<0.05. ^†^One haemodialysis patient with high baseline plasma concentrations of sucralose was excluded and not included in analysis with regards to sucralose, erythritol and S:E ratios.

#### Whole bowel permeability

**Figure 4:**
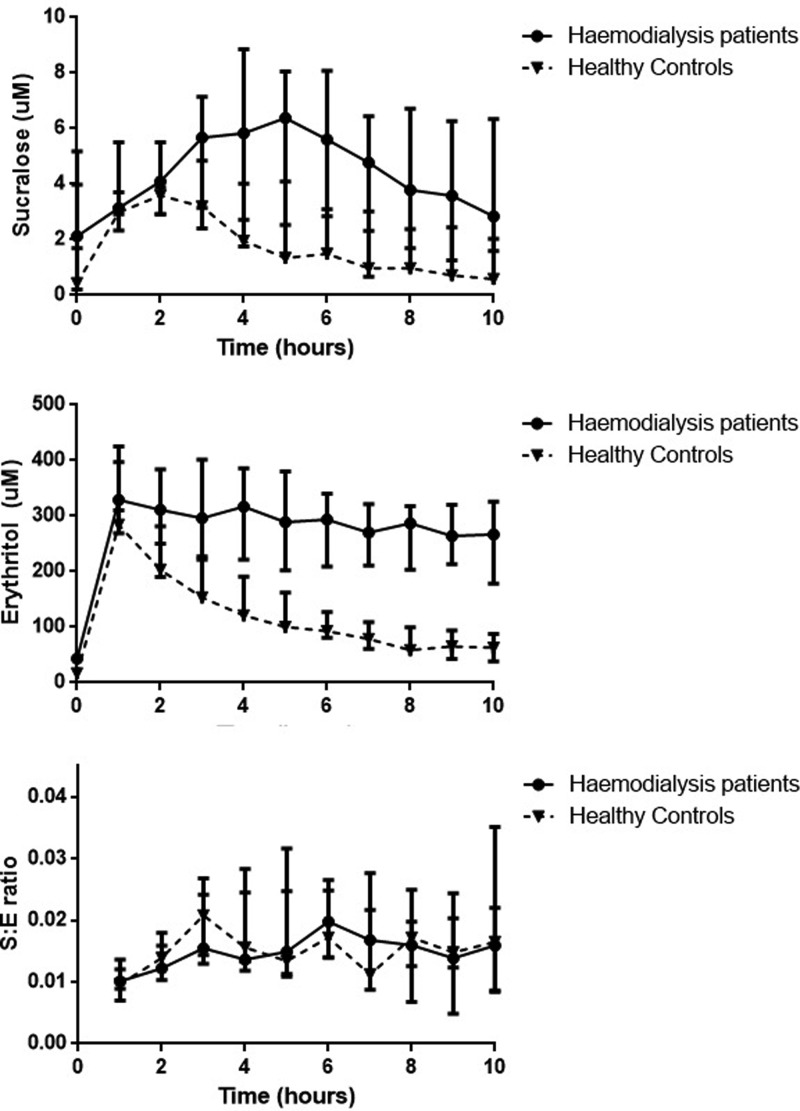
Plasma levels of sucralose, erythritol and S:E ratios in haemodialysis patients and healthy controls

One haemodialysis patient had very high baseline levels of sucralose and was excluded from whole bowel permeability analysis using sucralose and erythritol. Sucralose levels increased post-ingestion peaking at 2 h for healthy controls, for haemodialysis patients sucralose levels peaked at 5 h and the magnitude of peak levels reach were approximately two-fold greater compared with controls (7.09 vs. 3.59 μM, *P*=0.205). Erythritol levels peaked at 1 h and reached similar level of magnitude for both haemodialysis patients and healthy controls, however for haemodialysis patients, erythritol levels remained persistently elevated with no discernible reduction in levels for the whole study period, probably reflecting the lack of renal clearance. AUC for S/E ratios were not significantly different between haemodialysis patients and healthy controls (Figure 4 and Table 4).

The pharmacokinetics of these sugar probes are strongly affected by the lack of renal function as these sugars are predominantly removed by the kidneys. Use of ratios to compare intestinal permeability in haemodialysis patients with healthy controls may not be reliable (see ‘Discussion’ section).

### Relationship between level of residual kidney function and concentration of sugar probes

Due to the strong effect of renal function on the clearance of sugar probes, the relationship between residual renal function in haemodialysis patients with profiles of sugar probes was investigated. There was no significant correlation between residual urea clearance (KRU) and AUC for lactulose, rhamnose, sucralose or lactulose. Differences in AUC for all four sugar probes were compared between anuric HD patients and those with residual kidney function (Table 5), there were no significant differences in AUC for all sugar probes between HD patients with and without residual renal function.

**Table 5:** AUC for sugar probes in haemodialysis patients with and without residual renal function

	No residual renal function (anuric) [*n*=4]	Residual renal function present (KRU 1.1–3.2 ml/min) [*n*=6]	*P*
AUC Lactulose	16.6 [11.7–30.8]	12.6 [10.7–17.4]	0.394
AUC Rhamnose	566 [470.5–730.2]	541.2 [375.7–767.8]	0.67
AUC Sucralose	39.4 [26.7–54.1]	62.5 [21.3–98]	0.624
AUC Erythritol	2451.5 [2061.8–3202]	3482 [2373–3683]	0.327

Data shown are median and interquartile ranges.

## Discussion

This is the first study to evaluate intestinal permeability in haemodialysis patients and the acute effect of haemodialysis on intestinal permeability *in vivo*. Breakdown of intestinal barrier function and increased permeability is associated with a number of diseases such as inflammatory bowel disease and increasingly believed to be implicated in systemic inflammation in uraemic states [4,10,30]. Researchers have attempted to assess gut barrier function using several methods, although each method has its own limitations. Assessment of *in vitro* gut barrier function with the use of conventional histological methods does not permit measurement of changes in intestinal permeability and data collected from biopsies taken from a small area of the bowel may not be representative for the whole gut. Biomarkers of intestinal epithelial integrity have also been used such as blood endotoxin, d-lactate and intestinal fatty acid binding proteins, but these are all indirect measures of intestinal permeability and have their own limitations [reviewed in [31]].

The classical method to measure intestinal permeability *in vivo* is with the use of orally administered oligosaccharides first described in 1974 by Menzies [32], initially single-test substances of large molecular weights (such as lactulose and polyethylene glycol) were used, however the absorption of these substances could be influenced by pre- and post-mucosal factors other than intestinal permeability such as bacterial degradation, absorptive surface area, gastric dilution and gastrointestinal transit time, differences in systemic distribution of sugars and renal clearance. Thus, the test was modified by introducing a second smaller probe since this is thought to traverse the intestinal barrier freely independent of barrier loss but similarly affected by pre- and post-mucosal factors. A ratio of the urinary concentration of both probes provides a more accurate assessment of paracellular passage across the gut wall than a single probe [31]. By measuring the appearance of oligosaccharide probes in plasma rather than urine, we attempted to determine intestinal permeability in haemodialysis patients.

The haemodialysis procedure itself did not lead to acute changes in intestinal permeability. There were no significant differences in the L/R and S/E ratios between non-dialysis days and post-dialysis implying that the haemodialysis procedure itself does not appear to induce increased gut permeability. This is in contrary to previous suggestions that haemodialysis may exacerbate increased gut permeability due to observations of reduced intestinal perfusion from ultrafiltration during haemodialysis [17–19]. Thus, intestinal barrier dysfunction in kidney disease may be due to other causes such as gut oedema [5,33,34] and/or retained uraemic toxins. Previous studies have found that incubating human colonocytes in media containing pre-dialysis serum resulted in a marked drop in transepithelial electrical resistance indicating increased permeability. This was accompanied by loss of transcellular and intracellular protein constituents of the tight junction. The extent of epithelial barrier damage and dysfunction was reduced in cells exposed to serum obtained post-dialysis suggesting that intestinal barrier function is impaired by dialysable retained uraemic toxin(s) [15]. Future studies should be directed at the identification and maximising clearance of these permeability inducing toxins.

Several limitations may account for our findings. Firstly, patients selected for the present study were relatively young with little co-morbidity and modest ultrafiltration requirements. Increased intestinal permeability induced by haemodialysis may be detected in patients with greater co-morbidity, high ultrafiltration requirements or haemodynamic instability. Secondly, the study sampling period lasted only 10 h, previous studies have shown that elimination of some of the sugar probes such as sucralose and erythritol can last up to 48–72 h even in subjects with normal kidney function [35–37]. Intestinal transit time is delayed in uraemia, thus 10 h may have been insufficient to study colonic permeability. Additionally since, sugar probes (sucralose, lactulose) peaked late and did not return to baseline by the end of the study period, AUC for 10 h could have underestimated intestinal absorption.

Compared with healthy controls, plasma lactulose levels were significantly higher in haemodialysis patients although rhamnose levels were not significantly different. Plasma levels of lactulose progressively increased late into the study period and only started to reduce after 8 h. The peak levels of lactulose in haemodialysis patients were significantly higher than controls (approximately three-fold higher). Whereas for rhamnose, peak levels and AUC for haemodialysis patients were similar to healthy controls, although the high levels of rhamnose were sustained for a longer period in haemodialysis patients (Figure 1). This effect is likely to be due to reduced clearance from lack of renal function. Since both lactulose and rhamnose are primarily excreted by the kidney [38,39], it would be expected that the magnitude of rise of both sugar molecules in haemodialysis patients relative to healthy controls would be similar. However for lactulose, the degree of absorption by haemodialysis patients is significantly higher than healthy controls while there was no significant difference in plasma levels of rhamnose leading to a significantly higher L/R ratio. Although these findings suggest increased small bowel permeability in haemodialysis patients it is difficult to ascertain whether this reflects increased absorption of lactulose due to increased intestinal permeability or accumulation of lactulose due to lack of kidney function. Differences in absorption profiles of these two sugars may also contribute. Studies in humans show that rhamnose is rapidly absorbed after ingestion and further absorption does not occur beyond 1.5 h post-ingestion despite a significant amount of rhamnose remaining in the gut [40]. On the other hand, absorption of lactulose occurs more uniformly in the small intestine and can continue for up to 4 h after ingestion [41], which may lead to accumulation and progressively rising plasma lactulose levels in patients who lack kidney function.

Sucralose and erythritol resist bacterial degradation in the colon and were used to evaluate whole intestinal permeability. Both sucralose and erythritol are primarily excreted by the kidney, with the clearance rate of erythritol estimated to be approximately half the rate of creatinine [37,42], both are also known to undergo a small but currently unquantified amount of extra-renal metabolism [35,36]. In accordance with previous studies, sucralose and erythritol in healthy controls reached peak levels at approximately 1–2 h after ingestion [35–37]. Erythritol levels in haemodialysis patients peaked at a similar time scale and magnitude as healthy controls whereas in haemodialysis patients, peak sucralose levels occurred later in the study period (at approximately 5 h post-ingestion) and were two-fold greater than healthy controls. Similar to lactulose, the later peaking of sucralose levels is likely due to accumulation secondary to renal impairment. The S/E ratio in the two groups was not significantly different but the interpretation of this is complicated by the influence of kidney function [44]. The higher sucralose levels in haemodialysis levels may well reflect increased intestinal permeability but may also indicate the effect of other factors, particularly lack of kidney function.

In addition to the effect of kidney function, other factors may contribute to the differences in plasma oligosaccharide levels between haemodialysis patients and controls. Uraemia may induce delays in gastric emptying and reduce gut motility [43]. Haemodialysis patients were on a large number of medications (Table 2) including drugs that are known to affect gastrointestinal motility such as proton pump inhibitors and opiates. The effect of medications such as statins and phosphate binders that are frequently used by haemodialysis patients on intestinal permeability has not been studied in detail and it is unclear if medication use may have influenced the plasma levels of sugar probes in the present study. Hence, although we consider differences in renal clearance to be the major factor complicating the interpretation of our findings in relation to differences between haemodialysis patients and controls, there may well be other factors which need to be taken into account.

In summary, an accurate and convenient method of measuring intestinal permeability in haemodialysis patients remains elusive. Due to the dependence of sugar probes on kidney function for clearance, comparison of intestinal permeability between subjects with and without kidney disease is fraught with difficulties. Use of probes that are not removed by the kidney may overcome this problem but none of the currently available intestinal permeability probes are suitable from this perspective. The intestinal permeability measurement technique described in the present study may be used to measure gut permeability changes in response to an intervention or stimuli, although the need for a prolonged sampling period would make it difficult to apply in the clinical setting. However, it is important to recognise that although intestinal permeability assays have been used frequently in gastroenterology research, the mechanisms that determine oligosaccharide intestinal permeability may be different from that used by bacterial products and intestinal permeability determined by these methods may not correlate with clinically significant bacterial intestinal translocation [31,45]. In conclusion, this is the first study to measure intestinal permeability in dialysis patients *in vivo*, contrary to previous suggestions we did not detect any significant acute changes in intestinal permeability in relation to the haemodialysis procedure.

## Clinical perspectives

- The present study was conducted to determine if the haemodialysis procedure exacerbates gut permeability. Translocation of bacterial products through a hyper-permeable intestinal barrier is considered a potential source of systemic inflammation in haemodialysis patients. The cause of increased gut permeability in uraemia is not well understood, but it is widely postulated that haemodialysis exacerbates permeability due to large blood volume changes during treatment leading to mesenteric ischaemia however this has not been demonstrated *in vivo*.
- The present study showed that haemodialysis does not increase intestinal permeability acutely.
- The findings from the present study showed that increased intestinal permeability in haemodialysis patients is due to other factors rather than the haemodynamic changes that occur during haemodialysis treatment. These observations further our understanding of the mechanisms of increased intestinal permeability in uraemia.

## Supporting information

**Table S1:** Characteristics of patients with CRC and controls

## Abbreviations

AUC: area under curve
CKD: chronic kidney disease
LC-MS: liquid chromatography and mass spectrometry
L/R or L:R: lactulose:rhamnose ratio
S/E or S:E: sucralose:erythritol ratio
