# Supplementary material for: Acute haemodynamic changes during haemodialysis do not exacerbate gut hyperpermeability
Source: Biosci Rep. 2019 Apr 12;39(4):BSR20181704. doi: 10.1042/BSR20181704 (PMC6477914; doi:10.1042/BSR20181704)
Supplement: Supplementary file 1 [file bsr20181704_Supp1.pdf]

Table S1 Characteristics of patients with CRC and controls

| Parameters | CRC group (n=225) | Control group (n=200) | $\chi^2$-value | *p*-value |
|---|---|---|---|---|
| Age (years) | | | 6.018 | 0.111 |
| 20~40 | 7(3.00%) | 8(4.00%) | | |
| 41~60 | 101(44.89%) | 102(51.00%) | | |
| 61~80 | 112(49.78%) | 90(45.00%) | | |
| ≥81 | 5(2.22%) | 0(0.00%) | | |
| Gender | | | 0.461 | 0.497 |
| Male | 140(62.22%) | 118(59.00%) | | |
| Female | 85(37.78%) | 82(41.00%) | | |
| BMI (kg/$m^2$) | 25.84±6.55 | 25.79±6.83 | 0.077 | 0.939 |
| Smoking status | | | 0.058 | 0.810 |
| Yes | 111(49.33%) | 101(50.50%) | | |
| No | 114(50.67%) | 99(49.50%) | | |
| Alcohol consumption | | | 2.702 | 0.1 |
| Yes | 54(24.00%) | 35(17.50%) | | |
| No | 171(76.00%) | 165(82.50%) | | |
